# Effects of Exercise on Balance, Gait Speed, Quality of Life, and Symptom Relief Among Older Adults with Parkinson’s Disease: A Meta-Analysis of Randomized Controlled Trials (RCTs)

**DOI:** 10.3390/healthcare13172212

**Published:** 2025-09-04

**Authors:** Jeong-Hui Park, Tyler Prochnow, Matthew Lee Smith, Jung-Min Lee, Christina Amo

**Affiliations:** 1 School of Public Health, Texas A&M Health Science Center, 212 Adriance Lab Rd., College Station, TX 77843, USA; tprochnow@tamu.edu (T.P.); matthew.smith@tamu.edu (M.L.S.); 2 Center for Health Equity and Evaluation Research, Texas A&M University, College Station, TX 77843, USA; 3 Center for Community Health and Aging, Texas A&M University, College Station, TX 77843, USA; 4 Graduate School of Physical Education, Kyung Hee University (Global Campus), Giheung-gu, Yongin-si 17104, Gyeonggi-do, Republic of Korea; jungminlee@khu.ac.kr; 5 Sports Science Research Center, Kyung Hee University (Global Campus), Giheung-gu, Yongin-si 17104, Gyeonggi-do, Republic of Korea

**Keywords:** exercise intervention, Parkinson’s disease, RCTs, older adults

## Abstract

**Background:** Parkinson’s disease (PD) is a prevalent neurodegenerative disorder characterized by progressive impairments in balance, gait, and quality of life (QoL). Exercise interventions have emerged as complementary therapies, but their effectiveness remains unclear. This meta-analysis aimed to systematically evaluate the effects of exercise interventions on balance, gait speed, QoL, and symptom relief among older adults with PD. **Methods:** Following Cochrane Collaboration and PRISMA guidelines, randomized controlled trials (RCTs) published in peer-reviewed journals up to November 2023 were identified (*n* = 388) through PsycINFO, Scopus, PubMed, and Web of Science databases. Studies included adults aged ≥60 with PD, assessing exercise interventions compared to control conditions. Evidence quality was assessed using the Grading of Recommendations, Assessment, Development, and Evaluation (GRADE) system. Random-effects models with standardized mean differences (SMD) were used to analyze the effectiveness of exercise interventions on balance, gait speed, QoL, and symptom relief. **Results:** Eleven RCTs were analyzed. Exercise interventions showed no significant effect on balance (SMD = −0.06, *p* = 0.41), QoL (SMD = 0.06, *p* = 0.33), or PD symptom relief (SMD = 0.10, *p* = 0.45). However, a significant improvement in gait speed was observed (SMD = −0.90, *p* = 0.001). **Conclusions:** In older adults with PD, exercise significantly enhances gait speed only; evidence for balance, QoL, and symptom relief is non-significant, and interpretation is limited by between-study heterogeneity and small samples. Since various measurement tools across studies may have influenced the outcomes, future research should incorporate repeated measurements using more specific and consistent assessment tools to clarify the effectiveness of exercise interventions for older adults with PD.

## 1. Introduction

Parkinson’s disease (PD) stands as a progressive neurodegenerative disorder affecting the central nervous system [1]. It holds considerable global health significance, ranking as the second most prevalent neurological disorder [2], impacting approximately one million individuals in the United States and seven million globally [3,4]. PD is diagnosed in approximately 6 out of every 1000 North Americans with advanced age being a risk factor. In fact, those aged 80 years and older have a 400% increase in prevalence [5,6,7].

In the initial stages, PD manifests with mild symptoms predominantly affecting one side of the body [8]. Disease progression leads to motoneuron fluctuations and dyskinesia (e.g., tremors at rest, rigidity, and postural instability [9]). These progressions are associated with postural instability, heightened fall risk, gait freezing episodes, speech impairments, and difficulties swallowing [10]. These hallmarks of PD can impede essential functional activities like transferring, walking, and daily activities of living [11,12]; and contribute to reduced confidence in balance and gait, and an increased susceptibility to falls [13].

While some studies have demonstrated enhancements in specific functions, like balance, through virtual reality exercise interventions [14,15,16], others found no substantial disparities in gait ability, motor function, or overall QoL [17,18,19]. Given the escalating symptoms and prolonged duration of PD, motor impairments are anticipated to substantially impact the mental as well as physical well-being of affected individuals [20,21]. Previous researchers have documented unfavorable consequences of PD including amplified social isolation, diminished participation in leisure pursuits, and greater reliance on assistance for daily activities. These factors collectively contribute to a decline in the overall quality of life (QoL) for those with PD [22,23,24,25]. However, the question of whether therapeutic interventions, such as medication and exercise, lead to improvements in the QoL remains a topic of debate.

More specifically for exercise intervention, randomized controlled trials (RCTs) have yielded inconsistent outcomes across motor, functional, and mental health domains [26,27]. For example, a six-month, home-based, remotely supervised aerobic program improved motor skills, indicating symptom relief [26]. Similarly, a 24-week program of slow, mindful movement improved postural control, gait, and functional reach; and lowered fall incidence for three months [27]. By contrast, large pragmatic and behavior-change trials often produce non-significant results. In the PD REHAB trial, individualized physical and occupational therapy did not yield clinically meaningful gains in activities of daily living or health-related quality of life at 3–15 months [28]. The ParkFit trial, an intensive behavior-change program designed to increase day-to-day activity did not raise overall activity [29]. Likewise, a large, home-based fall-prevention program did not reduce repeat falls over six months [30].

Rigorous evaluations of exercise specifically in older adults with PD remain scarce. Additionally, trial populations often underrepresent older individuals or fail to report age-stratified outcomes, limiting the applicability to those most affected by multimorbidity, frailty, and fall risk. To address these gaps, the current study presents a preregistered systematic review and meta-analysis focused on adults aged 60+ years with PD. We comprehensively searched multiple bibliographic databases and trial registries from inception to the present, screened records in duplicate, and included randomized controlled trials of structured exercise delivered in clinical, community, or home settings. This study’s specific research questions (RQ) are as follows:

RQ #1: Does exercise intervention enhance balance and/or postural stability in older people with PD?
RQ #2: Does exercise intervention improve gait speed in older people with PD?
RQ #3: Does exercise intervention improve health-related QoL in older people with PD?
RQ #4: Does exercise intervention alleviate PD symptoms in older people with PD?

## 2. Materials and Methods

### 2.1. Study Design

This meta-analysis was conducted in accordance with the Cochrane Collaboration guide [31] and the Preferred Reporting Items for Systematic Reviews and Meta-Analyses (PRISMA) guidelines [32]; and was preregistered in the PROSPERO database (CRD420251109367).

### 2.2. Data Sources and Search Strategy

The current investigation employed a systematic methodology for the identification of relevant peer-reviewed studies up to 9 November 2023. A comprehensive search was conducted using electronic databases: PsycINFO, Scopus, PubMed, and Web of Science. The search terms used were as follows: (“physical activit*” OR “exercise*” OR “workout” OR “aerobic” OR “walking”) AND (“Parkinson’s disease” OR “Parkinson” OR “neuropsychological”) AND (“older adult*” OR “elder*” OR “senior citizen” OR “retire*” OR “geriatric*”). Keywords were grouped to ensure sensitivity/specificity: exercise domain (e.g., ‘exercis*’, ‘aerobic’, ‘walking’) to capture structured interventions; condition (‘Parkinson*’) to exclude non-PD populations; and age (‘older adult*’, ‘geriatric*’) to restrict to ≥60 years. All identified records were subsequently managed using Covidence [33].

### 2.3. Literature Review and Meta-Analysis Process

**Figure 1:**
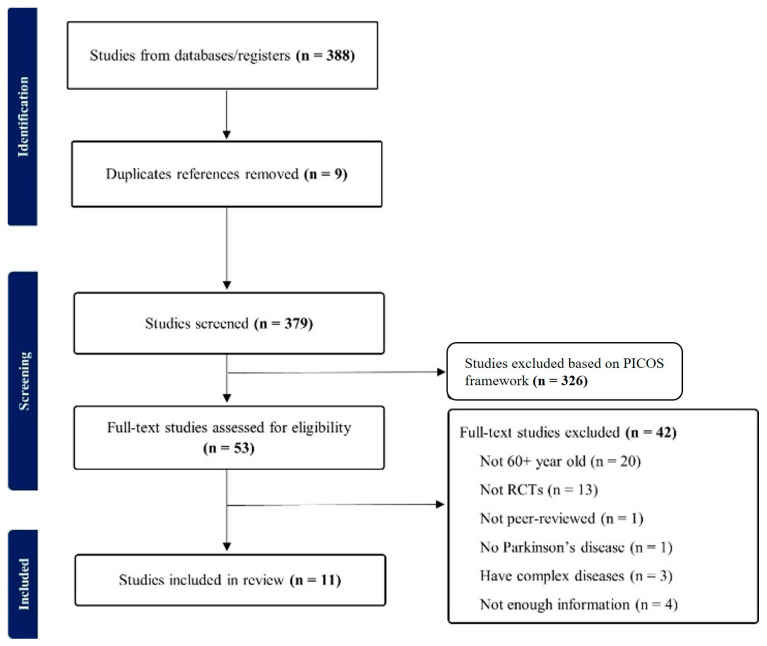
PRISMA flow diagram.

Study inclusion and exclusion criteria were established based on the Population, Intervention, Comparison, Outcome, and Study design (PICOS) framework. For a comprehensive overview and a visual representation of the selection process, Figure 1 presents the complete data and diagram following the PRISMA guidelines [32].

The inclusion criteria were as follows: (P) Population: participants were individuals aged 60 years or older who had a clinical diagnosis of PD. (I) Intervention: studies implemented structured exercise intervention programs. (C) Comparison: exercise interventions were compared with a control condition consisting of no intervention or standard care. (O) Outcome: quantitative outcomes assessed included balance, gait speed, QoL, and PD symptom relief. (S) Study design: only RCTs published in peer-reviewed English-language journals were included. Studies were excluded if they involved non-RCT designs, participants younger than 60 years, unavailable full-text articles, were published in languages other than English, or did not pertain specifically to structured exercise interventions for older adults with PD. Two independent reviewers conducted the screening and selection process, resolving any discrepancies through discussion and consensus or, when necessary, consultation with a third reviewer.

The initial search yielded a total of 388 articles, after which duplicate articles were excluded (*n* = 9), reducing the count to 379. Subsequently, the PICOS framework was used to exclude 326 articles during title and abstract screening. The remaining articles (*n* = 53) underwent a full-text assessment using an independent, double-rater system (IRR = 0.85). Discrepancies between the two reviewers were resolved through discussion and consensus or, if necessary, by consulting a third reviewer. During the full-text assessment, a total of 42 articles were excluded based on various criteria, including involving participants under 60 years of age (*n* = 20), lacking RCTs (*n* = 13), being commentaries or editorials (*n* = 1), encompassing participants without PD (*n* = 1), including individuals with complex medical conditions (e.g., dementia, falling, and urinary incontinence; *n* = 3), or lacking essential outcome values (means and/or standard deviations; *n* = 4); this study may have the potential for selection bias arising from this exclusion. The final sample consisted of 11 articles, which were then subjected to data extraction for meta-analysis (Table 1).

### 2.4. Quality and Bias Assessment

Two independent reviewers conducted a thorough assessment of bias risk within the selected studies. In this systematic review, the risk of bias in RCTs was evaluated using the second version of the Cochrane risk of bias tool for randomized trials (RoB2) [44]. The criteria under consideration encompassed the following aspects: (1) the randomization process, (2) deviations from the intended intervention, (3) missing outcome data, (4) the measurement of the outcome, and (5) the selection of reported results. Each of these criteria was categorized as either presenting a ‘low risk of bias,’ ‘some concerns,’ or ‘high risk of bias’.

### 2.5. Quality of Evidence

The quality of evidence was assessed using the Grading of Recommendations, Assessment, Development, and Evaluation (GRADE) framework [45]. According to GRADE, the evidence was classified into four categories (high, moderate, low, and very low) indicating accuracy of the estimated effect (compared to the actual effect). The criteria for the quality of evidence included: risk of bias (low, moderate, or high), inconsistency (considerable variability in effect sizes, minimal overlap between confidence intervals, and significant statistical heterogeneity among studies), indirectness (limitations or mismatches regarding population, intervention type, comparator conditions, or outcomes across included studies), imprecision (the lower or upper confidence limit exceeded 0.5 of the standardized mean difference in either direction), and publication bias (funnel plot asymmetry, selective reporting, or potential conflicts of interest [46,47]).

### 2.6. Effect Size and Direction

The present study computed standardized mean differences (Hedges g) from post-intervention means and SDs (or change scores when only those were available) using a random-effects model. Because outcome instruments differ in whether higher scores indicate improvement or impairment, we did not impose a single sign convention across all instruments. Instead, we report effects in the clinically native direction of each instrument and, for clarity, state which sign favors exercise in each figure caption: (1) Balance (i.e., BBS, Mini-BESTest; QoL: SF-36): more positive SMDs indicate greater improvement, (2) Gait speed (i.e., GAITRite velocity, gait velocity, DGI): more negative SMDs indicate greater improvement (i.e., faster gait/better performance), (3) QoL: more positive SMDs indicate greater improvement of quality of life, (4) Symptom Relief: for instruments where larger values reflect impairment (e.g., Unified Parkinson’s Disease Rating Scale [UPDRS], PDQ-39, GDS, HAM-D17, FES-I, and time-based TMT), more negative SMDs indicate greater improvement (i.e., lower symptom burden or better performance).

### 2.7. Statistical Analysis

The meta-analysis in this study was first conducted using the meta package in R 4.3.2 statistical software [48]. Given the variability in the assessment instruments for physical functioning (e.g., balance and gait speed), QoL, and symptoms across studies, we employed the standardized mean difference (SMD). Recognizing the potential diversity in participants, intervention types, and outcome measurement tools, we adopted a random effects model for the outcome analysis, as it was more suitable than a fixed model. To gauge between-study heterogeneity, we utilized Cochran’s Q test and I^2^ statistics. Potentially influential studies were identified using leave-one-out analyses and influence diagnostics (DFBETAS > |0.5| or studentized residuals > |2|) to flag outliers. A sensitivity analysis was conducted to evaluate the influence of individual studies on the overall effect. This study qualitatively assessed publication bias using a forest and funnel plot and quantitatively analyzed it through Egger’s linear regression tests. Effect size estimates with two-sided *p*-values below 0.05 were deemed statistically significant.

## 3. Results

### 3.1. Balance

**Figure 2:**
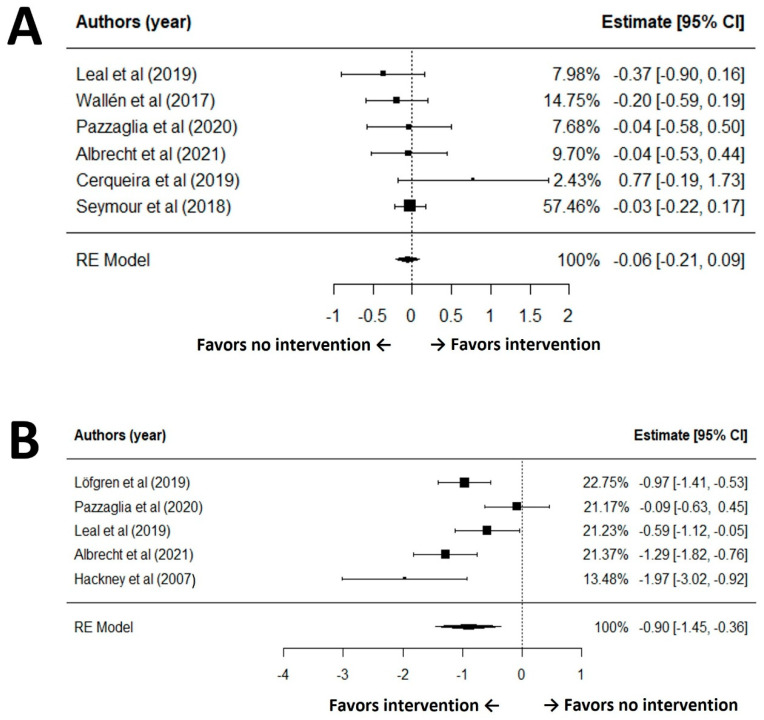
Forest plot for meta-analysis of balance (**A**) and gait speed (**B**) outcomes [30,34,37,38,39,40,42,49].

According to the GRADE of balance, the certainty of evidence for balance outcomes was moderate. Although the analysis had no issues with risk of bias, inconsistency, indirectness, or publication bias, the precision of the effect estimate was limited by the lack of statistically significant findings. Therefore, the quality of evidence was downgraded from high to moderate due to this imprecision. After excluding influential outliers (studies 2, 3, and 9) identified in sensitivity analyses, a random effects meta-analysis of the remaining six studies revealed no statistically significant effect of exercise interventions on balance among older adults with PD (SMD = −0.06, 95% CI: −0.21 to 0.09, *p* = 0.41) (Figure 2A). The heterogeneity among these studies was not significant (I^2^ = 0.0%, Q(5) = 4.81, *p* = 0.44) and the regression test for funnel plot asymmetry (Egger’s test) indicated no publication bias in the effect sizes for balance outcomes among the included studies (*p* = 0.76). The pooled effect size corresponds to a trivial change (|SMD| < 0.20) in balance, indicating no clinically meaningful benefit at the group level.

### 3.2. Gait Speed

Based on the GRADE assessment of gait speed, the certainty of evidence for gait speed outcomes was assessed as very low. While the included studies generally did not present serious concerns regarding risk of bias, inconsistency was observed due to substantial heterogeneity among studies (I^2^ = 78.0%, Q(4) = 15.80, *p* = 0.003). After excluding influential outliers (studies 4, 5, and 7) identified in sensitivity analyses, a random-effects meta-analysis of the remaining five studies demonstrated a statistically significant effect of the intervention on gait speed (SMD = −0.90, 95% CI: −1.45 to −0.36, *p* = 0.001) (Figure 2B). Although it showed high heterogeneity (I^2^ = 78.0%, Q(4) = 15.80, *p* = 0.003), the Egger’s test revealed no evidence of publication bias in the effect sizes for gait outcomes among the included studies (*p* = 0.50). For gait speed, the pooled SMD ~0.90 indicates a moderate-to-large improvement favoring exercise.

### 3.3. Quality of Life (QoL)

**Figure 3:**
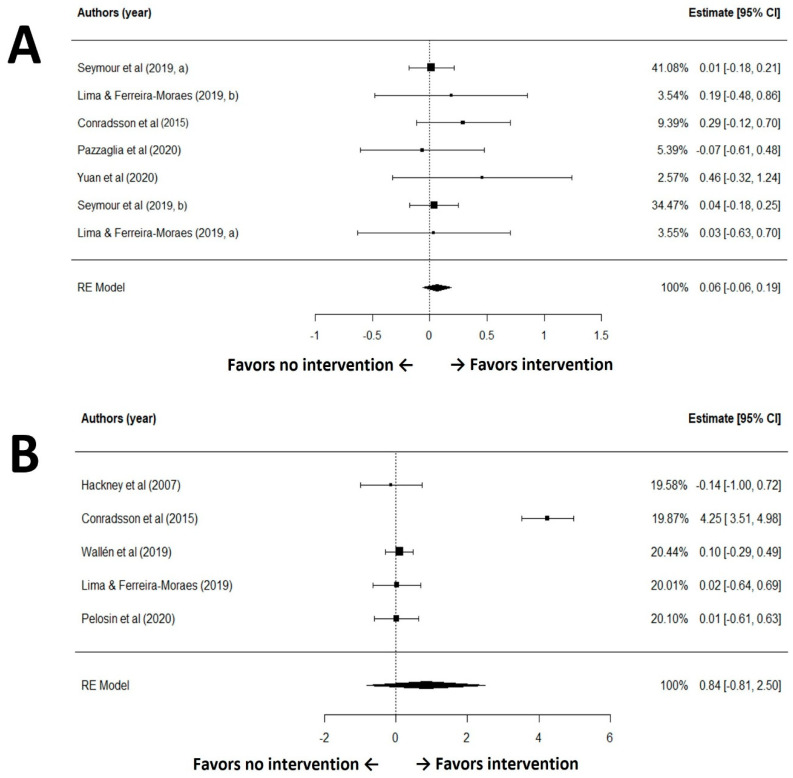
Forest plot for meta-analysis of quality of life (**A**) and Parkinson’s disease symptom (**B**) outcomes [30,35,36,38,40,41,42,43].

The GRADE rated the certainty of evidence for quality of life (QoL) outcomes as moderate. The included studies did not raise substantial concerns regarding risk of bias, inconsistency, indirectness, or publication bias. A random-effects meta-analysis of seven comparisons revealed no statistically significant difference in QoL scores between groups receiving exercise interventions and those receiving no exercise (SMD = 0.06, 95% CI: −0.06 to 0.19, *p* = 0.33; Figure 3A). The analysis demonstrated negligible heterogeneity among studies (I^2^ = 0.0%, Q(6) = 2.83, *p* = 0.83), and Egger’s test indicated no evidence of publication bias for QoL outcomes in the included studies (*p* = 0.20). For quality of life, the pooled magnitude (|SMD| < 0.20) is negligible and not consistent with a clinically meaningful group-level benefit.

### 3.4. Parkinson’s Symptom Relief

The GRADE rated the certainty of evidence for this outcome as moderate. The included studies did not raise substantial concerns regarding risk of bias, inconsistency, indirectness, or publication bias. A random-effects meta-analysis of five studies found no statistically significant difference between the intervention and control groups (standardized mean difference [SMD] = 0.10, 95% CI: −0.15 to 0.34, *p* = 0.45; Figure 3B). The analysis demonstrated no heterogeneity among studies (I^2^ = 0.0%, Q(4) = 0.24, *p* = 0.99), and Egger’s test indicated no evidence of publication bias (*p* = 0.20). Regarding symptom relief, the estimated effect remains trivial (|SMD| < 0.20); a clinically relevant improvement at the population level is therefore unlikely.

## 4. Discussion

This review aimed to determine whether structured exercise improves balance, gait speed, QoL, and PD symptoms among adults aged 60+ years with PD. We achieved this aim by assessing 11 RCTs. Overall, exercise produced a significant improvement in gait speed (RQ2), but not for balance (RQ1), QoL (RQ3), or global PD symptoms (RQ4). Also, gait speed results should still be interpreted cautiously given between-study heterogeneity in gait speed outcomes, variation in assessment tools and intervention content.

Exercise’s insignificant impact on balance does not align with recent studies demonstrating and enduring effects of balance and gait in long-term intervention for individuals with PD [35,39,46]. The studies included in our meta-analysis may dissent due to substantial variations in intervention designs and durations, ranging from 4 weeks to 12 months. Furthermore, Furthermore, recent network and conventional meta-analyses suggest that certain modes (e.g., exergaming, dance, rhythmical auditory exercise, aerobic training) can be efficacious for postural balance, TUG, and gait velocity, although comparative advantages across modalities are often modest and head-to-head evidence remains limited [47,50,51,52]. This study’s inclusion criteria may also have contributed: many published trials enroll mixed-age samples with earlier disease severity, whereas our meta-analysis targeted only 60 years and older. This age discrepancy may explain that exercise interventions effect balance less (or not at all) for those 60 and over. Subgroup evidence focused on older adults indicates that resistance training may preferentially improve Mini-BESTest, while aerobic training can better enhance UPDRS-III, gait velocity, and TUG, underscoring that age and prescription parameters may shape balance responsiveness [53].

The pooled effect for walking speed was statistically significant, indicating that exercise programs can improve gait velocity in older adults with PD. Similar conclusions are reported in recent studies showing beneficial effects of structured exercise on gait parameters (e.g., velocity and step length), even as the magnitude varies across regimens and study designs [52,54]. Several factors like differences in modality (e.g., aerobic training, treadmill, dance or tai chi) and dose (e.g., session frequency and program duration), measurement approaches (e.g., 10-m walk, GAITRite, Dynamic Gait Index), and assessment contexts (e.g., testing ON vs. OFF medication), as well as baseline severity and adherence can account for the observed variability [55]. Notably, network and conventional meta-analyses suggest that programs with adequate dosing and specific modalities (e.g., aerobic or treadmill-based training) tend to yield larger gains in walking speed, supporting a dose-response and modality-specific interpretation rather than a null effect [52,54]. Also, some physiological mechanisms plausibly account for selective gains in walking speed with exercise in PD. First, task-specific motor learning inherent to treadmill and overground walking practice refines step length and cadence, yielding faster velocity [56,57]. Second, external cueing, particularly rhythmic auditory stimulation, entrains gait timing via auditory-motor coupling and facilitates more automatic stepping, which increases speed [58]. Third, improvements in lower-limb power, especially plantarflex or strength and rate-of-force development for push-off, are associated with faster gait in PD and likely mediate part of the exercise effect on velocity [59]. Finally, aerobic conditioning achieved during ambulatory exercise supports higher steady-state walking speeds; exercise may also act synergistically with dopaminergic therapy to reduce bradykinesia during straight-ahead gait [57,60]. Nevertheless, to reduce heterogeneity, trials still need to standardize exercise dosing using full FITT parameters and harmonize gait speed assessment protocols, including test type, pace instructions, trial number, and medication state. Pre-registration of core outcome sets with minimal clinically important difference thresholds, detailed reporting of adherence and fidelity, and adequate duration and power are recommended.

In the older adults (60+ years) with PD cohort, the pooled effect of exercise on QoL was not statistically significant. Several factors may account for this null finding. First, QoL instruments differ in content and directionality (e.g., generic scales such as SF-36 and PD-specific measures like PDQ-39), which can dilute pooled effects when studies use mixed tools or target different domains (e.g., mobility vs. mood) (see instrument discussion in Materials and Methods). Second, prior meta-analyses in broader PD samples report heterogeneous QoL responses by exercise type and dose (e.g., aerobic, dance/martial arts, and ≥12-week programs showing greater improvements), suggesting that insufficient dose or non-optimized modalities within the older subgroup may attenuate effects [61]. Third, QoL in PD is strongly driven by non-motor symptoms like depression, anxiety, fatigue, and sleep problems whose burden often increases with age and may not be sufficiently modified by the primarily motor-focused interventions [62,63]. Finally, variability in intervention content, short follow-up, and small sample sizes likely reduced precision, consistent with other studies that find clear motor benefits of exercise but inconsistent QoL changes [52]. Collectively, these considerations support a cautious interpretation: demonstrable gains in QoL for older adults with PD may require longer-duration, non-motor–targeted, and domain-specific programs (e.g., integrating mood/sleep components) and the use of harmonized, PD-specific QoL measures. Future trials should stratify by exercise modality and dose, include components targeting non-motor determinants of QoL, and adopt consistent PD-specific QoL metrics (e.g., PDQ-39) to improve comparability across studies.

This systematic review and meta-analysis employed the UPDRS to quantify the influence of various therapeutic exercise interventions on PD symptoms. UPDRS evaluates both motor and non-motor symptoms, covering manifestations such as bradykinesia, rigidity, resting tremor, postural instability, anosmia, sleep disorders, psychiatric symptoms, cognitive impairment, autonomic dysfunction, fatigue, and pain [64]. However, pooled estimates indicated no statistically significant change in global motor symptom severity (UPDRS/MDS UPDRS) among older adults with PD. Several factors may account for this null finding. First, dose and modality are critical: meta-analyses in broader, not age restricted samples report small benefits that are greater with aerobic exercise (e.g., dance or tai chi) and adequate duration and frequency; whereas lesser doses (which are common in trials for older adults) tend to yield attenuated effects [52,54,65]. Second, measurement context can obscure change; studies combined UPDRS and MDS UPDRS, assessed participants in heterogeneous ON or OFF medication states, and often used brief follow up intervals. Observed differences may therefore fall below the minimal clinically important difference for MDS UPDRS Part III (approximately 3.25 points), resulting in statistically null or clinically trivial effects [66]. Third, heterogeneity among intervention content and guidance, confounds the dose–response adaptations of structured exercise interventions [54]. Taken together, these considerations warrant a cautious interpretation; although specific exercise prescriptions can improve motor performance in broader PD populations, demonstrable relief of global motor severity in older adults may require longer duration, higher dose, consistent medication-state assessments, and harmonized use of PD specific symptom scales.

Traditionally, PD treatment primarily relies on anti-Parkinsonian medications; however, prolonged use of medications is associated with adverse effects such as peak-dose dyskinesia, on-off phenomena, and wearing off [67]. Surgical interventions, such as deep brain stimulation employed to mitigate the physiological alterations in brain tissue induced by PD [68]; however, these interventions are characterized by their high costs, elevated risk of side effects [69], and the potential reoperation needs. Furthermore, non-motor symptoms in PD patients often exhibit limited responsiveness to conventional medical therapies [70], emphasizing the significance of complementary therapies, notably rehabilitation exercises. In this context, the consideration of long-term complementary therapies, such as rehabilitation exercises, becomes pertinent ion addressing non-motor symptoms related to QoL (e.g., fatigue, depression, and anxiety) in individuals with PD.

While PD has a higher incidence in the older population, previous studies have generally been conducted in middle-aged adults over 40 years of age with PD. However, our study is the first to specifically report on the effectiveness of exercise interventions on physical function, symptom relief, and QoL among older adults with PD. Thus, there are some limitations to our study. First, substantial heterogeneity was observed in the gait speed variable under consideration. This diversity was attributed to significant differences in the quality of the studies, the characteristics of the participants, and the nature of exercise treatments and routine activities outlined in each article. Therefore, for more nuanced insights like the publication year, exercise type, and participants’ characteristics, it is recommended that future investigations employ meta-regression analyses to scrutinize sub-groups. Second, the sample sizes within the literature reviewed for this study remain relatively modest. The segmentation of the study into two groups, based on the presence or absence of regular exercise intervention regardless of the specific intervention types (e.g., walking, balancing, aerobic, and complex exercises), resulted in a total of 11 distinct articles. Consequently, the distribution of articles across each evaluation index was limited, potentially compromising the statistical robustness of the tests conducted. Third, several full-text RCTs lacked variance estimates (means and SDs) or reported non-comparable statistics; consistent with our analytic plan, we did not impute missing variances or converted non-equivalent summaries, which may bias pooled effects toward more completely reported studies. To enhance the validity of findings, it is advocated that subsequent research endeavors, particularly those involving older populations, incorporate larger sample sizes and additional clinical papers focusing on exercise therapy interventions. Lastly, this study prespecified exploratory subgroup analyses by exercise modality, program duration, and session frequency, but did not conduct them because strata were sparse (often <3 studies) and dose parameters were inconsistently reported. Future research should pre-register and adequately power subgroup comparisons—by modality (e.g., aerobic, balance-focused, multimodal), duration, frequency, and intensity—to identify effect modifiers in older adults with PD. This approach would facilitate more comprehensive and conclusive insights into the efficacy of exercise interventions for this demographic.

## 5. Conclusions

This systematic review and meta-analysis offer valuable insights into the effects of exercise interventions, especially the gait speed of the geriatric population afflicted with PD. In adults aged 60+ with PD, structured exercise consistently improves gait speed. However, evidence for balance, QoL, and symptoms is inconclusive. Nevertheless, to delineate effects beyond gait, future trials should employ harmonized outcome definitions, standardized assessment tools, and adequate statistical power. From a clinical standpoint, these findings support the integration of tailored exercise programs as an adjunct to pharmacological care to enhance physical functioning, particularly gait speed, in older adults with PD. Embedding individualized rehabilitation within multidisciplinary, person-centered care pathways may enable more effective and personalized management for this population.

## Figures and Tables

**Table 1:** Literature review for RCTs study characteristics included in meta-analysis.

Author(s), Year	Title	EG (N)	CG (N)	Age	Exercise Type	Intervention	Outcome(s)	Outcome Tool(s)	Frequency (Time/Week)	Duration (Week)
Albrecht et al., 2021 [34]	Effects of a Highly Challenging Balance Training Program on Motor Function and Brain Structure in Parkinson’s Disease	34	31	70.4	Combined exercise	Balance training	Balance, Gait	Mini-BESTest, GAITRite	2	10
Conradsson et al., 2015 [35]	The Effects of Highly Challenging Balance Training in Elderly With Parkinson’s Disease: A Randomized Controlled Trial	47	44	73.3	Combined exercise	Balance and gait training	Balance, Gait, Symptom	Mini-BESTest, GAITRite, UPDRS	3	10
de Lima et al., 2019 [36]	Resistance training reduces depressive symptoms in elderly people with Parkinson disease: A controlled randomized study	17	16	67.2	Anaerobic exercise	Resistance training	Gait, Symptom, QoL	Walking speed, UPDRS, PDQ-39, HAM-D17	2	20
de Melo Cerqueira et al., 2020 [37]	Cognitive and motor effects of Kinect-based games training in people with and without Parkinson disease: A preliminary study	8	8	68.3	Combined exercise	Videogame training	Balance, Gait, QoL, Symptom	BBS, FOG-Q, PDQ-39, UPDRS	2	5
Hackney et al., 2007 [38]	Effects of tango on functional mobility in Parkinson’s disease: a preliminary study	9	10	66.4	Combined exercise	Dance and martial arts (Tai chi)	Balance, Gait, Symptom	BBS, Walking speed, FOG-Q, UPDRS	2	13
Leal et al., 2019 [39]	Low-volume resistance training improves the functional capacity of older individuals with Parkinson’s disease	27	27	65.1	Anaerobic exercise	Resistance training	Balance, Gait	TMT-G, TMT-B	2	24
Pazzaglia et al., 2020 [40]	Comparison of virtual reality rehabilitation and conventional rehabilitation in Parkinson’s disease: a randomized controlled trial	25	26	71.0	Combined exercise	Virtual reality training	Balance, Gait, QoL	BBS, DGI, SF-36	3	6
Pelosin et al., 2020 [41]	A Multimodal Training Modulates Short Afferent Inhibition and Improves Complex Walking in a Cohort of Faller Older Adults With an Increased Prevalence of Parkinson’s Disease	17	22	72.6	Combined exercise	Virtual reality training	Symptom	UPDRS	3	6
Seymour et al., 2019 [30]	Multicentre, randomized controlled trial of PDSAFE, a physiotherapist-delivered fall prevention program for people with Parkinson’s	183	211	72.0	Combined exercise	Home-based training	Balance, QoL	Mini-BESTest, FES-I, GDS	2	24
Wallén et al., 2018 [42]	Long-term effects of highly challenging balance training in Parkinson’s disease-a randomized controlled trial	51	49	73.1	Combined exercise	Balance and gait training	Balance, Gait, Symptom	Mini-BESTest, GAITRite, UPDRS	3	10
Yuan et al., 2020 [43]	Effects of interactive video-game-based exercise on balance in older adults with mild-to-moderate Parkinson’s disease	12	12	67.2	Combined exercise	Virtual reality training	Balance, QoL	BBS, SF-36, MFES	3	24

Abbreviations: RCTs, randomized controlled trials; EG: Experimental Group; CG: control group; M: mean; SD: standard deviation; QoL: quality of life; Mini-BESTest: Mini Balance Evaluation Systems Test; UPDRS: Unified Parkinson’s Disease Rating Scale; PDQ-39: Parkinson’s Disease Questionnaire; HAM-D17: Hamilton Depression Rating Scale; BBS: Berg Balance Scale, FOG-Q: Freezing of Gait Question; TMT: Trail Making Test; DGI: Dynamic Gait Index; SF-36: Short Form-36; FES-I: Falls Efficacy Scale; GDS: Geriatric Depression Scale; MFES: Modified Falls Efficacy Scale.

## Data Availability

The datasets used and/or analyzed during the current study are available from the corresponding author upon reasonable request.
